# TosR-Mediated Regulation of Adhesins and Biofilm Formation in Uropathogenic Escherichia coli

**DOI:** 10.1128/mSphere.00222-18

**Published:** 2018-05-16

**Authors:** Courtney L. Luterbach, Valerie S. Forsyth, Michael D. Engstrom, Harry L. T. Mobley

**Affiliations:** a Department of Microbiology and Immunology, University of Michigan Medical School, Ann Arbor, Michigan, USA; University of Kentucky

**Keywords:** UPEC, adherence, gene regulation, nonfimbrial adhesin

## Abstract

Uropathogenic Escherichia coli strains utilize a variety of adherence factors that assist in colonization of the host urinary tract. TosA (type one secretion A) is a nonfimbrial adhesin that is predominately expressed during murine urinary tract infection (UTI), binds to kidney epithelial cells, and promotes survival during invasive infections. The *tosRCBDAEF* operon encodes the secretory machinery necessary for TosA localization to the E. coli cell surface, as well as the transcriptional regulator TosR. TosR binds upstream of the *tos* operon and in a concentration-dependent manner either induces or represses *tosA* expression. TosR is a member of the PapB family of fimbrial regulators that can participate in cross talk between fimbrial operons. TosR also binds upstream of the *pap* operon and suppresses PapA production. However, the scope of TosR-mediated cross talk is understudied and may be underestimated. To quantify the global effects of TosR-mediated regulation on the E. coli CFT073 genome, we induced expression of *tosR*, collected mRNA, and performed high-throughput RNA sequencing (RNA-Seq). These findings show that production of TosR affected the expression of genes involved with adhesins, including P, F1C, and Auf fimbriae, nitrate-nitrite transport, microcin secretion, and biofilm formation.

**IMPORTANCE** Uropathogenic E. coli strains cause the majority of UTIs, which are the second most common bacterial infection in humans. During a UTI, bacteria adhere to cells within the urinary tract, using a number of different fimbrial and nonfimbrial adhesins. Biofilms can also develop on the surfaces of catheters, resulting in complications such as blockage. In this work, we further characterized the regulator TosR, which links both adhesin production and biofilm formation and likely plays a crucial function during UTI and disseminated infection.

## Abstract (precis)

Uropathogenic E. coli strains cause the majority of UTIs, which are the second most common bacterial infection in humans. During a UTI, bacteria adhere to cells within the urinary tract, using a number of different fimbrial and nonfimbrial adhesins. Biofilms can also develop on the surfaces of catheters, resulting in complications such as blockage. In this work, we further characterized the regulator TosR, which links both adhesin production and biofilm formation and likely plays a crucial function during UTI and disseminated infection.

## INTRODUCTION

Uropathogenic Escherichia coli (UPEC) strains are the primary cause of uncomplicated urinary tract infections (UTIs), a widespread public health issue, with approximately half of all women and one-fifth of men experiencing at least one UTI in their lifetime (1, 2). Most uncomplicated UTIs arise when bacteria from the intestine contaminate the periurethral area, traverse the urethra, and colonize the bladder, resulting in cystitis (3). In some cases, bacteria ascend the ureters and infect the kidneys, resulting in pyelonephritis. From the kidney, bacteria are capable of crossing the epithelial and endothelial barriers to spread via the bloodstream, which in severe cases can lead to urosepsis and death (4).

Compared to commensal E. coli, UPEC genomes encode numerous accessory proteins, including adhesins, toxins, and siderophores, which provide a fitness advantage during colonization of the host urinary tract system (5–7). Many of these virulence genes reside on large regions of horizontally acquired DNA, termed pathogenicity-associated islands (PAIs) (8, 9). Indeed, the pyelonephritis isolate CFT073 contains 13 genomic islands (GIs) that account for nearly one-fifth of the genome (10–12). Of these GIs, seven have been confirmed as PAIs (9, 10, 13, 14). We have previously shown that PAI-*aspV* harbors the *tosRCBDAEF* operon, which encodes the repeat-in-toxin (RTX) family member TosA (14, 15). RTX protein family members are frequently encoded on large open reading frames and can perform a range of functions, including pore and biofilm formation and adherence to host cells (16–18). TosA functions as an RTX nonfimbrial adhesin and can adhere to human kidney epithelial cells (15). Additionally, production of TosA occurs during both murine and human UTIs, and a mutant lacking *tosA* was attenuated for colonization of the bladder and kidneys in the murine model of UTI (14, 19). The TosCBD proteins mediate production and export of TosA, while TosE and TosF have an unknown regulatory function associated with suppression of motility (15, 20). The *tos* operon is regulated by TosR and the global regulatory proteins H-NS and Lrp (21). Our laboratory has previously shown that TosR functions as both an activator and repressor of the *tos* operon (20, 21). We found that low levels of TosR correlated with increased TosA production, while high levels of TosR inhibited TosA production (21).

TosR is a member of the PapB family of transcriptional regulators, which includes the fimbria-associated regulators PapB and FocB (20, 22–25). PapB and FocB bind as oligomers to AT-rich DNA motifs to mediate positive and negative regulation of the *pap* and *foc* operons, which encode the UPEC-associated P and F1C fimbriae, respectively (22, 23, 26). Pyelonephritis-associated pili, or Pap, bind Gal(α1-4)Gal moieties of the P-blood group antigen located on kidney cells and erythrocytes (27–29). F1C fimbriae bind glycosphingolipids found on kidney cells and also promote biofilm formation in the commensal E. coli isolate Nissle 1917 (30–32). Cross-regulation between fimbrial operons has been extensively studied. For example, PapB and FocB mediate the cross talk between the *pap*, *foc*, and *fim* operons, the latter encoding type 1 fimbriae (23, 26, 33–36). While PapB and FocB share over 80% amino acid sequence identity, they differ in their functions as regulators of fimbrial operons. In particular, FocB is a positive regulator of the *pap* operon and a dual regulator of the *foc* operon, PapB is a dual regulator of the *pap* operon and a repressor of the *foc* operon, and both FocB and PapB are negative regulators of the *fim* operon (24, 26, 35, 37, 38).

TosR shares only 27.7% amino acid sequence identity with PapB, but may share a similar function in regulating fimbrial expression based on predicted structural homology (20). Indeed, we have previously shown that TosR binds upstream of the *pap* operon and suppresses production of PapA, the major structural subunit of P fimbria (21). However, it is unclear if TosR regulates additional fimbrial genes, as observed with PapB and FocB, or additional nonfimbrial genes. Thus, to further define TosR-mediated effects on gene expression, in particular on other adhesin genes, we ectopically expressed *tosR*, collected mRNA, and performed high-throughput RNA sequencing (RNA-Seq). We discovered that TosR significantly affects gene expression of multiple functional gene categories, including adhesins, biofilm formation, microcins, and nitrite-nitrate transport. Specifically, when *tosR* was overexpressed, we observed dramatic upregulation of the *auf* operon, encoding Auf fimbriae, and downregulation of the *pap* and *foc* operons. UPEC isolates are more likely to encode Auf fimbriae, and production of Auf fimbriae occurs *in vivo* during murine UTIs (39, 40). We also observed that *tosR* overexpression led to increased Congo red and calcofluor white binding, and this phenotype was more robust in a mutant deficient in expression of the *auf* operon. Additionally, we observed that induction of *tosR* increased biofilm formation in lysogeny broth (LB) and human urine. Thus, our study shows the depth of TosR-associated regulation in a global network connecting genes encoding adhesins and other biofilm-promoting factors important in persistence and fitness during UTIs (7, 41).

## RESULTS

### Induction of *tosR* results in differential expression of both fimbrial and nonfimbrial genes.

To identify genes affected by TosR, we performed RNA-Seq on mRNAs derived from E. coli CFT073 harboring either pBAD-*tosR-*His_6_ or pBAD empty vector, because *tosR* is poorly expressed *in vitro* (15). Bacteria were cultured at 37°C in LB with aeration to mid-logarithmic growth, mRNAs were extracted, and RNA-Seq was performed. RNA sequence reads ranged from 10 to 15 million per sample, with 98.8 to 99.5% of these reads mapping to sequences in the reference E. coli CFT073 genome. Additionally, we excluded genes with variable or low expression by applying a cutoff requiring at least 3 of our counts per million (CPM) mapped reads for a given gene to be greater than 2. In total, overexpression of *tosR* resulted in the differential expression of 200 genes (123 upregulated and 77 downregulated) with a log_2_ fold change (FC) greater than or equal to ±1.5. The top 25 genes upregulated and downregulated (log_2_ FC, 10.1 to −6.2) in response to *tosR* overexpression are noted in Table 1 and Table 2, respectively.

**TABLE 1:** Top 25 genes upregulated in response to *tosR* overexpression^a^

Gene name	Gene locus^b^	Protein function	Log_2_ FC	*P* value	FDR
*tosR*	C_RS26215, c0359	PapB family transcription factor	10.1	8.3E−50	2.7E−46
c4594	C_RS21660, c4594	Uncharacterized protein	7.0	5.4E−47	8.6E−44
c0092	NA, c0092	Uncharacterized protein	6.6	2.3E−19	5.6E−17
*aufF*	C_RS19920, c4208	Auf fimbrial chaperone	6.6	8.6E−15	1.2E−12
c4924	C_RS23275, c4924	Putative hippuricase	6.6	2.1E−21	6.1E−19
*aufC*	C_RS19935, c4212	Auf fimbrial usher	5.6	1.5E−21	4.7E−19
*aufD*	C_RS19930, c4210	Auf minor fimbrial subunit	5.5	5.8E−16	1.1E−13
*aufB*	C_RS19940, c4213	Auf fimbrial chaperone	5.4	7.8E−22	2.8E−19
*aufA*	C_RS19945, c4214	Auf fimbrial major subunit	5.3	4.5E−32	3.6E−29
*yqiL*	C_RS18015, c3791	Yqi fimbrial subunit	5.3	8.3E−23	3.8E−20
*yfcV*	C_RS13680, c2884	Yfc fimbrial adhesin	5.2	4.4E−23	2.3E−20
c4423	C_RS20890, c4423	Uncharacterized protein	5.1	6.3E−15	9.2E−13
*pitB*	C_RS17665, c3724	Phosphate transporter	4.9	1.0E−18	2.2E−16
*aufE*	C_RS19925, c4209	Auf fimbrial minor subunit	4.4	3.2E−14	4.1E−12
*yicP*	C_RS21630, c4589	Adenine deaminase	4.4	4.6E−20	1.2E−17
c0325	C_RS01495, c0325	Uncharacterized protein	4.4	1.0E−14	1.4E−12
*tosC*	C_RS01650, c0360	TolC homolog	4.3	6.0E−19	1.4E−16
*efuD*	C_RS01485, c0322	Oligogalacturonide transporter	4.2	1.7E−23	1.1E−20
*yjjQ*	C_RS25720, c5444	Putative transcriptional regulator	3.7	2.9E−11	2.7E−09
*efuE*	C_RS01490, c0323	Exopolygalacturonate lyase	3.6	4.0E−22	1.6E−19
c2408	C_RS11410, c2408	Uncharacterized protein	3.5	7.3E−14	8.3E−12
c3178	NA, c3178	Uncharacterized protein	3.4	5.5E−15	8.3E−13
c1936	C_RS09090, c1936	F9 fimbrial major subunit	3.3	2.5E−12	2.8E−10
c0435	C_RS02025, c0435	Uncharacterized protein	3.3	7.1E−11	6.0E−09
*tsx*	C_RS23130, c4894	Nucleoside-specific channel	3.3	1.0E−10	8.1E−09

a NA, not available; FC, fold change; FDR, false-discovery rate.

b Gene locus tags contain the current NCBI annotation and the discontinued NCBI annotation, respectively.

**TABLE 2:** Top 25 genes downregulated in response to *tosR* overexpression^a^

Gene name	Gene locus^b^	Protein function	Log_2_ FC	*P* value	FDR
*narK*	C_RS07865, c1684	Nitrite extrusion protein 1	−6.2	5.2E−38	5.5E−35
*sdiA*	C_RS11040, c2330	Transcription factor	−3.6	2.8E−18	5.6E−16
c3655	C_RS17360, c3655	Antigen 43, autotransporter adhesin	−3.2	2.3E−05	7.7E−04
*narX*	C_RS07855, c1682	Histidine kinase	−2.7	3.6E−14	4.4E−12
*papH2*	C_RS24515, c5187	P fimbrial minor subunit	−2.7	1.6E−11	1.5E−09
*yhcS*	C_RS18950, c3998	Transcription factor	−2.5	5.5E−09	3.6E−07
*focC*	C_RS05825, c1241	F1C fimbrial chaperone	−2.4	4.9E−10	3.5E−08
*yffB*	C_RS14240, c2998	ArsC protein family reductase	−2.4	7.0E−09	4.4E−07
*sfaD*	C_RS05820, c1240	F1C fimbrial minor subunit	−2.4	4.9E−10	3.5E−08
*papB*	C_RS17045, NA	P fimbriae regulatory protein	−2.3	9.7E−11	8.0E−09
*papB2*	C_RS24525, NA	P fimbriae regulatory protein	−2.2	1.4E−09	9.4E−08
*yeiC*	C_RS12810, c2701	Pseudouridine kinase	−2.2	2.5E−03	4.2E−02
*papF2*	C_RS24485, c5180	P fimbrial minor subunit	−2.2	2.0E−08	1.2E−06
*papA2*	C_RS24520, c5188	P fimbrial major subunit	−2.2	9.4E−08	4.8E−06
*hybA*	C_RS17710, c3733	Hydrogenase-2 subunit	−2.1	8.9E−05	2.5E−03
*papH*	C_RS17035, c3591	P fimbrial minor subunit	−2.1	1.2E−06	4.9E−05
*focG*	C_RS05840, c1244	F1C fimbrial minor subunit	−2.0	3.5E−07	1.6E−05
*pmbA*	C_RS25180, c5333	Microcin B17 peptidase	−2.0	7.0E−09	4.4E−07
*focH*	C_RS05845, c1245	F1C fimbriae adhesin	−2.0	2.7E−07	1.3E−05
c1246	C_RS05850, c1246	F1C-associated phosphodiesterase	−2.0	2.0E−07	9.8E−06
*ynjE*	C_RS10200, c2158	Sulfurtransferase	−1.9	1.2E−05	4.3E−04
*focF*	C_RS05835, c1243	F1C fimbrial minor subunit	−1.9	4.8E−06	1.8E−04
*narL*	C_RS07850, c1681	Nitrate-nitrite response regulator	−1.9	2.7E−05	8.7E−04
*papF*	C_RS17005, c3584	P fimbrial minor subunit	−1.9	5.7E−07	2.6E−05
*focA*	C_RS05815, c1239	F1C fimbrial major subunit	−1.9	9.4E−07	4.0E−05

a NA, not available; FC, fold change; FDR, false-discovery rate.

b Gene locus tags contain the current NCBI annotation and the discontinued NCBI annotation, respectively.

**FIG 1:**
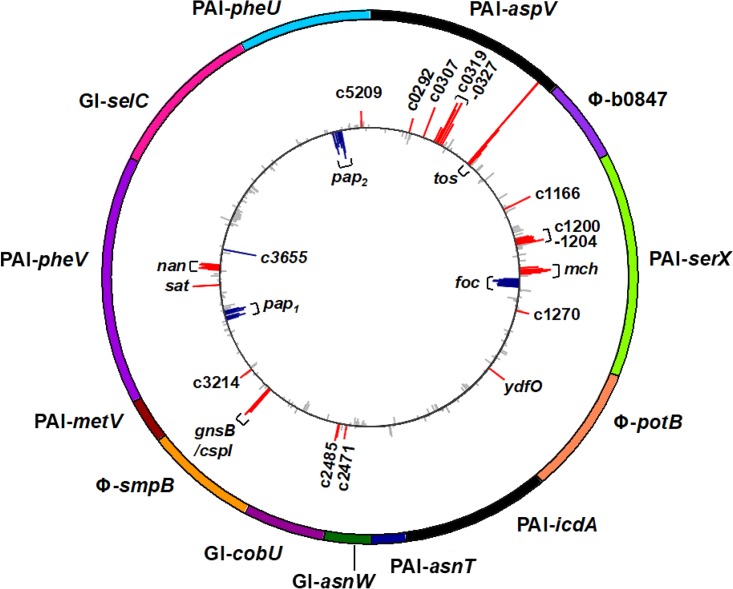
Overexpression of *tosR* affects expression of genes located on genomic islands. Shown is a schematic of genomic islands (GIs) and pathogenicity islands (PAIs) present in the CFT073 genome (outer circle). Colored segments indicate the length of each labeled DNA region. Each bar (inner circle) represents the log_2_ fold change for each gene present within the island, with red bars indicating a log_2_ fold change of ≥1.5, blue bars indicating a log_2_ fold change of ≤−1.5, and gray bars representing genes that were not differentially regulated. Clusters of red (upregulated) and blue (downregulated) bars are indicative of differential expression of whole operons in response to *tosR* overexpression.

E. coli genetic diversity is often mediated by horizontal gene transfer of large GIs, which frequently carry genes with accessory functions that are advantageous for host colonization, pathogenesis, or immune evasion (10, 12, 42, 43). The UPEC isolate CFT073 contains 13 GIs, with 7 being confirmed as PAIs (10, 14). We mapped the genomic locations of genes differentially expressed following overproduction of TosR to determine whether these genes are preferentially localized to GIs (Fig. 1). We found that a larger percentage of these genes, 68 out of 945 (7.2%), are located on GIs compared to the rest of the genome: 132 out of 4,476 (2.9%; *P* < 0.0001 by two-tailed Fisher’s exact test). In CFT073, the majority of GIs differ in G+C content compared to the rest of the genome (50.5%) (10). Since TosR is predicted to bind AT-rich sequences, we determined the association between the number of genes differentially regulated following induction of *tosR* and the total G+C content of the GI (see Table S1 in the supplemental material) (10, 21). We found that GIs with higher A+T content were more likely to harbor genes differentially expressed following TosR overproduction, with the exception of GI-*selC*, which did not have any genes affected by TosR-mediated regulation. Additionally through *in silico* analysis, we identified an AT-rich motif enriched in the upstream regions of differentially expressed genes (44.9%; *n =* 129) compared to nondifferentially expressed genes (11.5%; *n =* 52) (see Fig. S1 and Table S2 in the supplemental material).

10.1128/mSphere.00222-18.1 FIG S1  Putative TosR binding consensus sequence. Multiple Em for Motif Elicitation software (MEME version 4.12.0, http://meme-suite.org) was used to identify a 29-bp consensus sequence enriched in the upstream regions of a subset of differentially regulated genes (44.9%; *n =* 129) relative to nondifferentially regulated genes (11.5%; *n =* 52). The 36-bp TosR binding site (5′-ATAACAATAATATCTATAATATAGATATTATCTGCA) was used as a template for discriminative motif discovery within 450-bp DNA sequences located upstream of the translational start ATG of the individual gene or the operon. Download FIG S1, PDF file, 0.1 MB. Copyright © 2018 Luterbach et al. 2018 Luterbach et al. This content is distributed under the terms of the Creative Commons Attribution 4.0 International license.

10.1128/mSphere.00222-18.6 TABLE S1  Characteristics of CFT073 genomic islands harboring differentially regulated genes identified by RNA-Seq. Download TABLE S1, PDF file, 0.1 MB. Copyright © 2018 Luterbach et al. 2018 Luterbach et al. This content is distributed under the terms of the Creative Commons Attribution 4.0 International license.

10.1128/mSphere.00222-18.7 TABLE S2  Presence of a putative TosR binding consensus sequence upstream of differentially regulated genes. Download TABLE S2, PDF file, 0.2 MB. Copyright © 2018 Luterbach et al. 2018 Luterbach et al. This content is distributed under the terms of the Creative Commons Attribution 4.0 International license.

### TosR induces expression of multiple genes within the *tos* operon.

**FIG 2:**
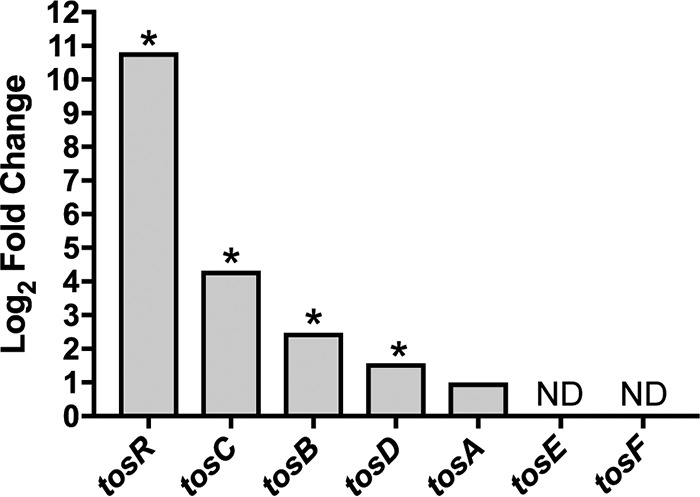
TosR-mediated induction of the *tos* operon. RNA-Seq demonstrates that TosR promotes expression of the *tos* operon. Each bar represents the log_2_ fold change in mRNA transcript levels of gene expression of CFT073 carrying pBAD-*tosR*-His_6_ compared to CFT073 carrying pBAD as an empty vector control. ND, no data (i.e., genes that did not return a sufficient number of sequence reads for analysis). *, log_2_ FC ≥ |± 1.5| and false discovery rate (FDR) < 0.05.

While TosR is both a positive and negative regulator of *tos* expression, we have previously shown by immunoblotting that the level of *tosR* induction used in the cells to derive mRNA for our RNA-Seq study results in an increase in TosA production (20, 21). Therefore, we predicted that overproduction of TosR would induce expression of the *tos* operon. As we expected, expression of the *tos* operon as determined by RNA-Seq revealed upregulation of *tosCBD* (log_2_ FC, 1.5 to 4.3). However, we did not observe a statistically significant change in *tosA* expression (log_2_ FC, 0.99), and insufficient reads for *tosE* and *tosF* precluded detection of differential gene expression (Fig. 2).

### TosR mediates differential expression of nonfimbrial genes.

Induction of *tosR* resulted in the differential expression of multiple nonfimbrial genes encoding proteins that participate in nitrate-nitrite transport, microcin production, quorum sensing, fucose metabolism, and the transport of metabolites and nucleosides, as well as many with uncharacterized functions. *narK* was the most downregulated gene (log_2_ FC, −6.22) identified using RNA-Seq in response to overexpression of *tosR* (Table 2). *narK* is transcribed with the *narKGHJI* operon and encodes a nitrate-nitrite transporter involved with the uptake of nitrate and excretion of nitrite (44–46). *narGHJI* genes encode subunits of a nitrogen reductase, which reduces nitrate to nitrite (46). The expression of *narGHJI* genes trended toward downregulation but was not statistically significant. Nitrate serves as an electron acceptor during anaerobic respiration, and *narK* has been previously identified as a fitness factor in UPEC F11 during murine UTI (47). Additionally, *narX* (log_2_ FC, −2.7) and *narL* (log_2_ FC, −1.9), part of the *narXLQ* operon, were also within the top 25 downregulated genes identified by RNA-Seq (Table 2). NarL and NarQ function as a two-component regulator that senses nitrate availability and subsequently induces expression of *narK* (48–50). It is unclear if TosR directly binds the promoters of the *narKGHJI* or *narXLQ* operons to mediate repression. Additionally, our RNA-Seq study assayed TosR-mediated regulation under *in vitro* conditions; therefore, the impact of TosR-mediated regulation of *nar* genes during UTI requires further investigation.

RNA-Seq also identified upregulation of all five genes of the *mchBCDEF* operon (log_2_ FC, 1.8 to 3.0), encoding microcin H47, located within PAI-*serX* (10). Microcins are small antibacterial peptides produced by bacteria that target the same or related species (51, 52). Microcin H47 is a known UPEC virulence factor, binds catechol receptors, and targets the ATP synthase for bactericidal activity (53–55). The ability to eliminate susceptible bacteria may provide an advantage to pathogenic strains during colonization (56). Upregulation of *mchBCDEF* has been observed during human and murine UTIs and during growth in human urine, which further underscores the importance of *tosR*-mediated gene regulation during UTI (10, 57).

### Overproduction of TosR affects expression of the *pap*, *foc*, and *auf* fimbrial operons.

**FIG 3:**
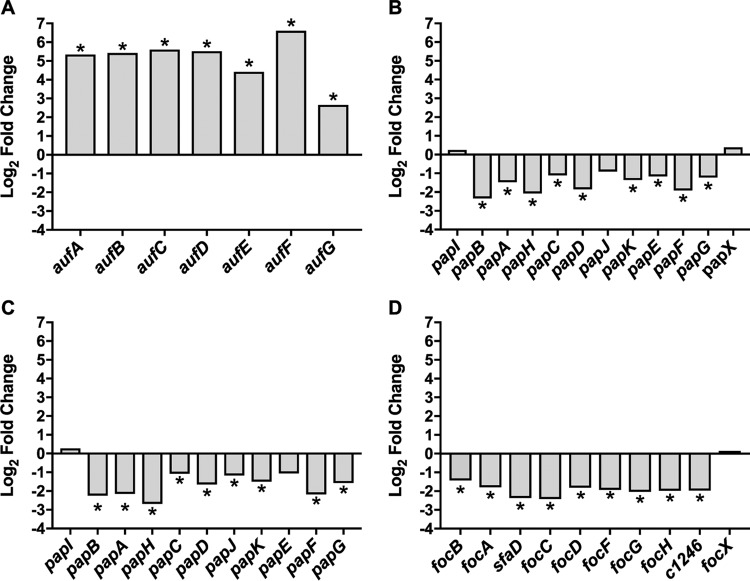
*tosR* overexpression leads to differential expression of fimbrial operons. (A to D) Data from RNA-Seq showing the log_2_ fold change in abundance of mRNA transcript levels compared between CFT073 carrying either pBAD or pBAD-*tosR*-His_6_ for each gene within the *auf* (A), *pap1* (B), *pap2* (C), and *foc* (D) fimbrial operons. NS, differences in gene expression are not significant. *, log_2_ FC ≥ |± 1.5| and false discovery rate (FDR) < 0.05.

The CFT073 genome encodes 12 distinct fimbriae, including 10 of the chaperone-usher family and 2 putative type IV pili (58). TosR shares predicted structural homology with PapB and FocB, both of which participate in fimbrial cross talk, and is therefore predicted to also regulate other fimbriae (20). Consistent with this prediction, RNA-Seq indicated that TosR regulates multiple operons encoding fimbriae. We observed significant upregulation of the *auf* operon (*aufABCDEFG*) (log_2_ FC, 2.6 to 6.6), which encodes Auf fimbriae (Fig. 3A). In contrast, we observed downregulation of the *pap1* (log_2_ FC, −2.3 to 0.38), *pap2* (log_2_ FC, −2.7 to −1.1), and *foc* (log_2_ FC, −2.4 to 0.15) operons (Fig. 3B to D). The CFT073 genome harbors two copies of the *pap* operon, designated *pap1* and *pap2*, both of which encode P fimbriae (58).

While we did identify additional differentially regulated fimbria-encoding genes within the *fim*, *yqi*, *F9*, *yad*, *yeh*, *yfc*, and *mat* operons, the majority of the genes associated with these operons were either not differentially regulated or were excluded due to mapped reads below the CPM cutoff value (see Fig. S4 in the supplemental material). Type 1 fimbriae, encoded by the *fim* operon, bind to mannose-containing glycoproteins located on epithelial cells within the lower urinary tract and are a virulence factor for E. coli during colonization of the urinary tract (59, 60). We observed a decrease in *fimA* (log_2_ FC, −1.8), encoding the fimbrial subunit, and a modest but statistically significant decrease in *fimB* (log_2_ FC, −1.2), encoding a recombinase that catalyzes the inversion of the *fim* regulatory switch (61). We did not identify significant differences in the gene expression of additional *fim* genes, which is not surprising since *fim* genes are poorly expressed during culture under aerated conditions (62, 63).

### Validation of differentially expressed fimbrial genes.

**FIG 4:**
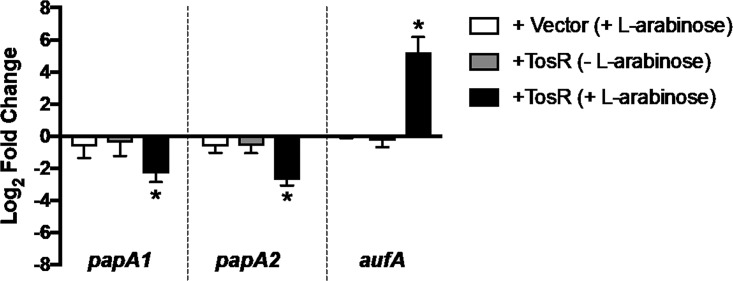
Overexpression of *tosR* represses *papA* expression and induces *aufA* expression. qPCR was performed, and bars represent the average (*n =* 3) log_2_ fold change in mRNA levels between CFT073 expressing pBAD-*tosR*-His6 (+TosR) and CFT073 expressing pBAD (+Vector) compared to an uninduced empty vector control. Data are normalized to the housekeeping gene *gapA*. Error bars represent standard deviation, and statistical significance was determined using Student’s *t* test. *, *P* < 0.05.

To validate our RNA-Seq results, we performed qPCR using primers specific to the fimbrial genes *papA1*, *papA2*, and *aufA* to compare log_2_ fold changes in gene expression between CFT073 carrying pBAD-*tosR*-His_*6*_ and CFT073 carrying pBAD. Strains were cultured in a manner that replicated our RNA-Seq experiment. We observed identical trends in gene expression compared to our RNA-Seq results. Specifically, we observed upregulation of *aufA* (log_2_ FC, 5.2) and downregulation of *papA1* and *papA2* (log_2_ FC, −2.3 and −2.7, respectively) (Fig. 4). We did not observe any changes in the gene expression of *papA1*, *papA2*, or *aufA* when comparing the wild type with the Δ*tosR* mutant using qPCR, and this may be due to poor *in vitro* expression of *tosR* (see Fig. S2 in the supplemental material). However, we found that overexpression of *tosR* decreased attachment to T24 human bladder epithelial cells, and this phenotype was abrogated in a mutant deficient in *auf* expression (see Fig. S3 in the supplemental material).

10.1128/mSphere.00222-18.2 FIG S2  Loss of *tosR* does not affect expression of *papA1*, *papA2*, or *aufA*. qPCR was performed, and bars represent the average (*n =* 2) fold change in mRNA levels compared between CFT073 and the Δ*tosR* mutant. Data are normalized to the housekeeping gene *gapA*. Error bars represent standard deviation, and statistical significance was determined using Student’s *t* test (*P* < 0.05). NS, differences in gene expression are not significant. Download FIG S2, TIF file, 1.9 MB. Copyright © 2018 Luterbach et al. 2018 Luterbach et al. This content is distributed under the terms of the Creative Commons Attribution 4.0 International license.

10.1128/mSphere.00222-18.3 FIG S3  Overexpression of *tosR* decreases attachment to T24 bladder epithelial cells. Shown is attachment of CFT073 carrying pBAD-*tosR*-His_6_ or the Δ*auf* mutant carrying either pBAD or pBAD-*tosR*-His_6_ to T24 human bladder epithelial cells. Bacteria were grown until the mid-logarithmic phase, induced with 10 mM l-arabinose, and then incubated with confluent bladder epithelial cells for 1 h at 37°C with 5% CO_2_. After incubation, bladder cells were washed three times with sterile phosphate-buffered saline (PBS) and lysed with 0.04% Triton X-100 for 20 min, and bacterial cells were enumerated on LB agar. Attachment levels of CFT073 carrying pBAD were set to 100%. Each bar represents the mean (*n =* 6). Error bars represent the standard deviation, and statistical significance was determined using Dunnett’s multiple-comparisons test. *, *P* < 0.05. Download FIG S3, PDF file, 0.3 MB. Copyright © 2018 Luterbach et al. 2018 Luterbach et al. This content is distributed under the terms of the Creative Commons Attribution 4.0 International license.

10.1128/mSphere.00222-18.4 FIG S4  Overexpression of *tosR* affects the expression of multiple fimbrial genes. (A to D) RNA-Seq data representing the log_2_ fold change in mRNA between CFT073 carrying either pBAD or pBAD-*tosR*-His_6_ for genes within the (A) type 1, (B) F9, (C) Yeh, (D) Yqi, (E) Yad, (F) Yfc, and (G) Mat operons. ND, no data (i.e., genes do not return a sufficient number of sequence reads for analysis). *, *P* < 0.05. Download FIG S4, PDF file, 0.1 MB. Copyright © 2018 Luterbach et al. 2018 Luterbach et al. This content is distributed under the terms of the Creative Commons Attribution 4.0 International license.

### **TosR induces curli**-**associated genes.**

**FIG 5:**
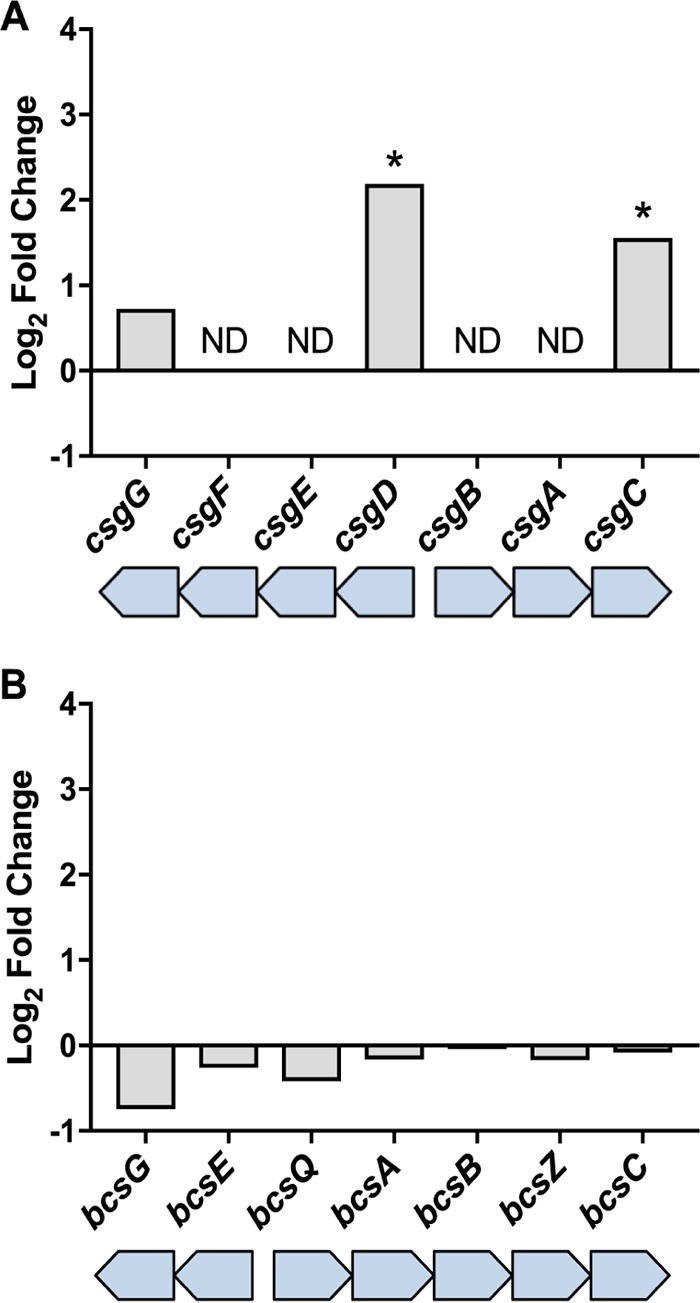
TosR increases expression of genes encoding curli, but not genes for cellulose production. Data from RNA-Seq show the log_2_ fold change in abundance of mRNA transcript levels compared between CFT073 carrying either pBAD or pBAD-*tosR*-His_6_ for each gene within the *csgD* and *bcsA* gene clusters. ND, no data (i.e., genes did not return a sufficient number of sequence reads for analysis). *, log_2_ FC ≥ |± 1.5| and false discovery rate (FDR) < 0.05.

Curli, amyloid-like fibers, assist in UPEC adherence to human uroepithelial cells and participate in the structural development of biofilms (64–66). Curli regulatory and structural genes are carried by the divergently expressed *csgBAC* and *csgDEFG* operons (67, 68). In response to *tosR* overexpression, we identified upregulation of two *csg* genes: *csgD* (log_2_ FC, 2.2) and *csgC* (log_2_ FC, 1.6) (Fig. 5A). *csgD* encodes a transcriptional regulator that induces the expression of *csgAB* encoding CsgA, the main curli fiber subunit, and CsgB, which mediates nucleation of CsgA (67, 69, 70). CsgC inhibits toxic intracellular amyloid formation by interfering with CsgA oligomerization (71, 72). We were unable to determine if *csgA*, *csgB*, *csgE*, and *csgF* were differentially expressed as they were excluded from the final RNA-Seq analysis due to low CPM values.

### *tosR* overexpression increases Congo red and calcofluor white binding.

**FIG 6:**
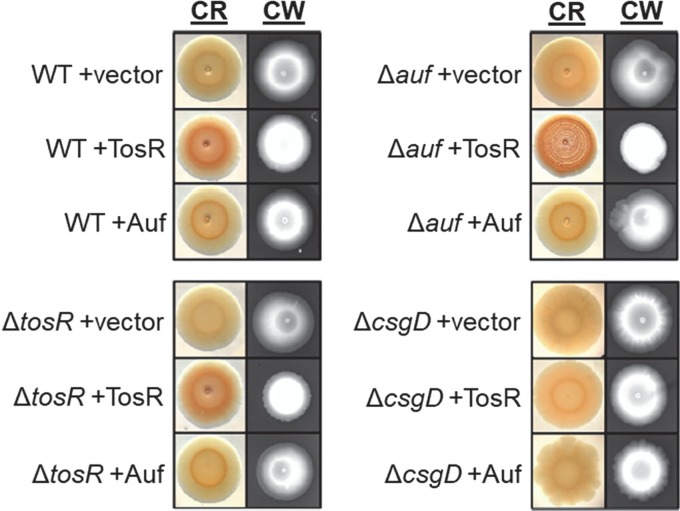
TosR overproduction increases binding to Congo red and calcofluor white. Data from Congo red (CR) and calcofluor white (CW) binding assays are shown comparing the CFT073 wild-type and Δ*tosR*, Δ*auf*, and Δ*csgD* mutant strains harboring either pBAD (+vector), pBAD-*tosR*-His_6_ (+TosR), or pBAD-*aufABCDEFG* (+Auf) after 48 h of incubation at 30°C. CR binding was visually determined as an increase in RDAR (rough, dry, and red) morphology, and CW binding was determined as an increase in fluorescence in the presence of UV light. Representative images from three independent experiments are shown.

Bacteria producing curli and/or cellulose will bind Congo red on YESCA plates (see Materials and Methods), which can result in an RDAR (red, dry, and rough) phenotype (73, 74). Previous studies have shown that expression of the *auf* operon was elevated in E. coli during biofilm formation, although a function for Auf in biofilm formation has not yet been determined (75, 76). Since overproduction of TosR resulted in an increase in *auf* expression, as well as *csgD*, a known regulator of curli biosynthesis, we investigated the contributions of TosR and Auf to Congo red binding. We did not observe any phenotypic differences between the Δ*tosR* mutant and wild type, but induction of *tosR* in the CFT073 wild-type and Δ*tosR* and Δ*aufABCDEFG* mutant strains led to an increase in Congo red binding compared to an empty vector control (Fig. 6). Interestingly, when TosR was overproduced in the Δ*aufABCDEFG* background, we observed a more pronounced RDAR phenotype compared to *tosR* overexpression in the wild type or in the *tosR* mutant, suggesting that Auf fimbriae are interfering with RDAR formation in the presence of high levels of TosR. Furthermore, deletion or overexpression of the *auf* operon did not have any apparent effect on Congo red binding. Loss of *csgD* abrogated Congo red binding and the RDAR phenotype, supporting that TosR-mediated regulation of *csgD* is contributing to increased amyloid formation.

CsgD also positively regulates the *bcsGE* and *bcsQABZC* operons involved in the production and export of cellulose, a secreted polysaccharide that functions as a structural component in the formation of biofilms (70, 77). To assess cellulose production, we performed a binding assay using the fluorescent cellulose-binding dye calcofluor white. We did not observe any difference in calcofluor white binding between the *tosR* mutant and wild type. However, induction of *tosR* in CFT073 wild-type and Δ*tosR* and Δ*aufABCDEFG* mutant strains increased calcofluor white binding, observed as an increase in fluorescence (Fig. 6). Additionally, overexpression of *tosR* in a *csgD* mutant did not increase binding of calcofluor white, suggesting that the presence of both CsgD and TosR is necessary for elevated binding of calcofluor white. Our RNA-Seq analysis did not identify any *bcs* genes as being differentially regulated in response to induction of *tosR* (Fig. 5B).

### TosR overproduction promotes biofilm formation in LB and human urine.

**FIG 7:**
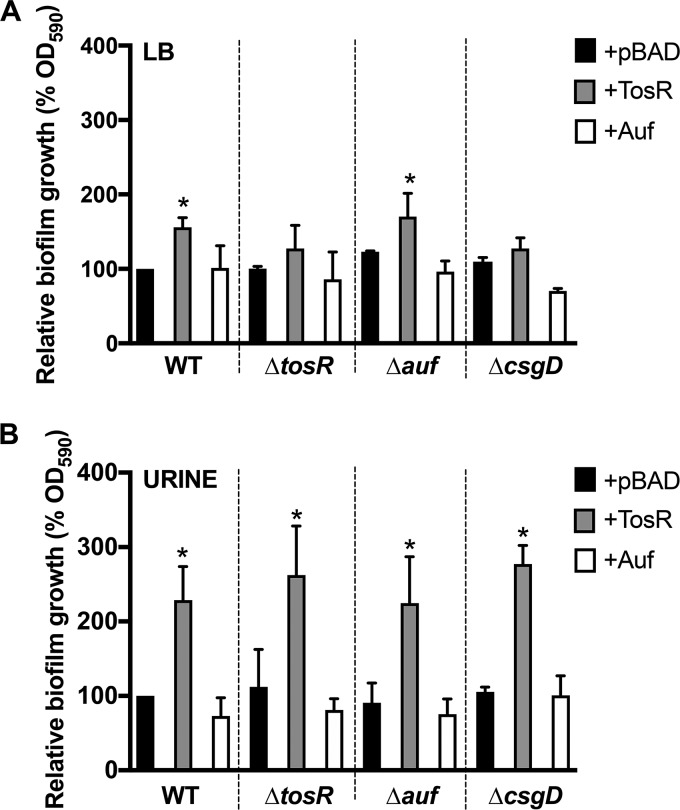
Overexpression of *tosR* increases biofilm formation in salt-free LB and human urine. Biofilm formation was measured in (A) salt-free LB or (B) pooled human urine in the CFT073 wild-type and Δ*tosR*, Δ*auf*, and Δ*csgD* mutants harboring either pBAD (empty vector control [+pBAD]), pBAD-*tosR*-His_6_ (+TosR), or pBAD-*aufABCDEFG* (+Auf). Biofilm growth was assessed using crystal violet and normalized to the induced wild type carrying pBAD (WT + pBAD). Each bar represents the mean absorbance from three biological replicates, with error bars showing standard deviation. Statistically significant differences between strains were determined using Dunnett’s multiple-comparison test. *, *P* < 0.05.

Biofilms are sessile bacterial communities that mediate cell-cell adherence as well as attachment to biotic and abiotic surfaces (78, 79). Since amyloid fibers and cellulose are two components that contribute to the complex formation of biofilms, we next investigated if increased expression of *tosR* would translate to increased biofilm formation. Therefore, we compared biofilm formation between the CFT073, Δ*tosR*, and Δ*aufABCDEFG* strains harboring pBAD, pBAD-*tosR*-His_6_, or pBAD-*aufABCDEFG*. We did not observe any differences in biofilm formation between the *tosR* mutant and wild type. However, we did observe a significant increase in biofilm formation, determined by increased retention of crystal violet, when *tosR* was overexpressed in both salt-free LB (Fig. 7A) and human urine (Fig. 7B) that was not due to an increase in biofilm inhabitants (see Fig. S5 in the supplemental material). We did not see any significant change in biofilm formation upon deletion or overexpression of the *auf* operon. Additionally, overexpression of *tosR* in the Δ*tosR* and Δ*csgD* mutants did not result in a statistically significant change in biofilm formation in salt-free LB but did increase biofilm formation in human urine.

10.1128/mSphere.00222-18.5 FIG S5  TosR does not affect levels of biofilm inhabitants. Shown are colony-forming units (CFU) of adherent (A) or total (adherent and nonadherent) (B) bacteria incubated statically in LB medium for 24 h at 37°C. To enumerate adherent bacteria, unbound cells were removed by washing twice with sterile 1× PBS. The remaining bacteria and matrix components were resuspended in sterile PBS using vigorous pipetting and then plated on LB agar with ampicillin and incubated at 37°C to enumerate viable counts. Removal of bacteria and matrix components was confirmed by crystal violet staining compared to an empty control well. Download FIG S5, PDF file, 0.3 MB. Copyright © 2018 Luterbach et al. 2018 Luterbach et al. This content is distributed under the terms of the Creative Commons Attribution 4.0 International license.

## DISCUSSION

Using RNA-Seq, our study further characterizes the function of the transcriptional regulator TosR in UPEC. In total, when TosR was overproduced, we identified 200 genes that were differentially expressed (123 upregulated and 77 downregulated). Based on structural homology to members of the PapB protein family, TosR is predicted to bind AT-rich sequences and mediate regulation by modulating the local nucleoid structure, similar to the mechanism of the nucleoid-structuring proteins Lrp and H-NS (21, 23, 80–82). Indeed, PapB has been shown to compete against H-NS and Lrp transcriptional silencing of the *pap* operon, and TosR is predicted to similarly antagonize the H-NS- and Lrp-mediated regulation of the *tos* operon (21, 23, 83). Overproduction of TosR resulted in elevated expression of *tosCDB* of the *tos* operon, but we did not see any significant upregulation of *tosAEF*. Previous work has shown via immunoblotting that expression of *tosR in trans* in the strain CFT073 results in a significant increase in TosA production compared to an empty vector control (21). However, high concentrations of TosR, as was also used in our study, resulted in only a slight increase in TosA production compared to the wild type and may explain why *tosA* gene expression trended toward upregulation but was not statistically significant.

PapB family members frequently participate in regulatory cross talk between fimbrial operons, and our previous work identified TosR as a negative regulator of the *pap* operon (20, 21, 26, 35). Our current RNA-Seq study supports these conclusions as we observed downregulation of the *foc*, *pap1*, and *pap2* operons, as well as upregulation of the *auf* operon in response to overexpression of *tosR*. The *auf* operon is more prevalent in uropathogenic than fecal E. coli strains (39). However, cochallenge infections between wild-type CFT073 and an isogenic mutant lacking the *auf* operon did not demonstrate Auf fimbriae as an important UPEC fitness factor during murine UTI (40). Interestingly, *aufA* is poorly expressed in urine samples collected from human UTI, but *aufDEG* was previously shown to be upregulated (1.8- to 2.5-fold) in the asymptomatic bacteriuria E. coli isolates 83972 and CFT073 during culture under biofilm-promoting conditions in human urine (75, 84). Therefore, Auf fimbriae may contribute more toward UPEC pathogenesis during catheter-associated UTIs, where biofilm formation on urinary catheters promotes a more persistent and severe infection (85, 86). As there is little information regarding regulation of the *auf* operon, to our knowledge, TosR is the first known regulator associated with this operon. While it is unclear whether TosR directly or indirectly promotes *auf* expression, we were able to identify a shared AT-rich motif that was enriched in the upstream regions of genes differentially regulated following *tosR* overexpression and may represent putative TosR binding sites (87–89).

The *tos* operon is more prevalent in UPEC isolates (~25 to 30%) than fecal E. coli strains (11%) (19, 20). As we have shown that induction of *tosR* affects the expression of multiple UPEC-associated fitness factors in CFT073, these results are likely broadly applicable to other UPEC strains carrying the *tos* operon. However, our RNA-Seq results represent genes affected by TosR induction during *in vitro* culture in LB, which may not comprehensively identify the genes regulated by TosR during infection. Therefore, additional gene expression studies under other growth conditions, such as human urine or *in vivo* studies, would add to our understanding of the impact of TosR on gene expression during pathogenesis.

Our study reveals that overproduction of TosR increases Congo red and calcofluor white binding, as well as biofilm formation in salt-free LB and human urine. We were able to show that Congo red and calcofluor white binding was dependent on the presence of *csgD*, encoding a transcriptional regulator of curli and cellulose production (67, 74, 90). We found that the loss of *tosR* did not affect Congo red binding, calcofluor white binding, or biofilm formation compared to the wild type. This may be due to limited native expression of *tosR* or indicate the presence of a compensatory regulatory mechanism. Interestingly, deletion of the *auf* operon promoted a more robust RDAR phenotype when TosR was overproduced in the Congo red binding assay but did not have any effect on TosR-mediated biofilm formation. We also found that overexpression of *tosR* decreased attachment to T24 human bladder epithelial cells, and this phenotype was abrogated in the *auf* mutant. Therefore, it may be that Auf fimbriae sterically inhibit the function of other adhesins or that deletion of *auf* affects the expression of genes encoding other adhesins or biofilm-related genes. As well, production of Auf fimbriae may affect the formation, secretion, or localization of biofilm components and the contribution of Auf to biofilm formation may be context dependent or require additional factors not present under our tested *in vitro* conditions. Indeed, overproduction of type 1, P, and F1C fimbriae prevents autoaggregation by the autotransporter protein Ag43, which is involved in cell-to-cell adhesion (91, 92). Therefore, deletion of the *auf* operon may impact biofilm development through an unknown mechanism.

Additionally, the absence of CsgD did not affect TosR-mediated biofilm formation when cultured in human urine, suggesting that additional regulatory or structural factors account for TosR-mediated biofilm formation, which is not surprising considering construction of biofilms is a complex association of curli, cellulose, fimbrial and nonfimbrial adhesins, flagella, colonic acids, and other exopolysaccharides (93–96). Therefore, expanded testing of these phenotypes under different culture conditions would improve our understanding of TosR-mediated regulation of *csgD*. Nevertheless, our results reveal for the first time that TosR-mediated gene regulation is part of a global gene network linking the regulation of adhesins and biofilm formation, and future studies should be designed to investigate TosR-mediated gene regulation during murine UTIs.

## MATERIALS AND METHODS

### Bacterial strains and media.

E. coli CFT073 was isolated from the blood and urine of a patient with acute pyelonephritis (11). Strains were cultured at 37°C with aeration in either lysogeny broth (LB; 10 g/liter tryptone, 5 g/liter yeast extract, 0.5 g/liter NaCl), LB without NaCl (salt-free LB; 10 g/liter tryptone, 5 g/liter yeast extract), or filter-sterilized pooled human urine. Urine was collected from at least 3 healthy female volunteers, pooled, filter sterilized, and stored at −20°C. Urine collection was performed as approved by the University of Michigan Institutional Review Board (HUM00004949). The following antibiotic concentrations were used when appropriate: ampicillin, 100 µg/ml; and kanamycin, 25 µg/ml. l-Arabinose (10 mM) was added to the medium to induce expression from the pBAD promoter when applicable.

### Construction of mutants and complementation.

The strains and plasmids used for this study are listed in Table S3 in the supplemental material, while the primers used are listed in Table S4. E. coli CFT073 mutants were constructed by recombineering (97). In brief, to construct the Δ*aufABCDEFG* mutant, a kanamycin resistance cassette was PCR amplified from pKD4 using EasyA polymerase (Agilent) and primers Δ*auf*KO_f and Δ*auf*KO_r and transformed into CFT073 expressing the λ Red recombinase system genes on the temperature-sensitive plasmid pKD46. Transformants were plated on LB agar with kanamycin and incubated overnight at 37°C. Deletion of the *aufABCDEFG* operon was confirmed by PCR using primers *auf*_screen_f and *auf*_screen_r. The Δ*csgD* mutant was constructed in a similar manner, but using the primers Δ*csgD*KO_f and Δ*csgD*KO_r, and the Δ*tosR* mutant was previously constructed (20).

10.1128/mSphere.00222-18.8 TABLE S3  Bacterial strains and plasmids used in this study. Download TABLE S3, PDF file, 0.1 MB. Copyright © 2018 Luterbach et al. 2018 Luterbach et al. This content is distributed under the terms of the Creative Commons Attribution 4.0 International license.

10.1128/mSphere.00222-18.9 TABLE S4  Primers used in this study. Download TABLE S4, PDF file, 0.1 MB. Copyright © 2018 Luterbach et al. 2018 Luterbach et al. This content is distributed under the terms of the Creative Commons Attribution 4.0 International license.

pBAD-*tosR*-His_6_ harboring *tosR* under the control of the arabinose-inducible *ara*BAD promoter was previously engineered (20). To generate pBAD-*auf*, the CFT073 *aufABCDEFG* operon was PCR amplified using EasyA polymerase (Agilent) and primers pBAD_auf_f and pBAD_auf_r. The resulting PCR product was digested with NcoI and KpnI (New England Biolabs) and ligated into pBAD-*myc*-HisA using T4 DNA ligase (New England Biolabs). The resulting construct was transformed into E. coli TOP10, and transformants were selected on LB agar containing ampicillin and verified by DNA sequencing. Plasmids were isolated and transformed into electrocompetent CFT073 or isogenic mutants and selected on LB agar with ampicillin.

### RNA isolation and sequencing.

E. coli CFT073 cells carrying either pBAD or pBAD-*tosR*-His_6_ were cultured overnight in biological triplicates in LB medium containing ampicillin. Cultures were diluted 1:100 into fresh LB medium containing 10 mM l-arabinose and ampicillin and cultured at 37°C with aeration. A 400-µl sample was collected at an optical density at 600 nm (OD_600_) of 0.46 to 0.96 and stabilized by the immediate addition of 800 µl of RNAprotect (Qiagen). Cells were then lysed with 0.2 µM lysozyme in TE (10 mM Tris-Cl, 1 mM EDTA, pH 8.0) for 5 min at room temperature, and total RNA was extracted using the RNeasy minikit (Qiagen). DNA contamination was eliminated by treatment with Turbo DNase (Thermo Fisher). Depletion of rRNA was accomplished with the Ribominus transcriptome isolation kit (Thermo Fisher) followed by ethanol precipitation. A stranded library was prepared using a ScriptSeq kit (Illumina) using the manufacturer’s recommended protocols. Each sample was tagged with a unique 6-nucleotide barcode for multiplexing. The products were purified and enriched by PCR to create the final cDNA library, which was checked for quality and quantity by TapeStation (Agilent) and qPCR using Kapa’s library quantification kit for Illumina sequencing platforms (Kapa Biosystems). Six samples were sequenced per lane on a 50-cycle single-end run on a HiSeq 2500 (Illumina) in high-output mode using version 4 reagents. cDNA reads were aligned to the CFT073 genome (NCBI GenBank accession no. NC_004431.1) by the Bioinformatics Core of the University of Michigan Medical School, and the program SPARTA was used for quality control analysis and calculation of differential gene expression, presented as log_2_ fold change (FC) (58, 98). Compositional biases between libraries were eliminated using trimmed means of *M*-values (TMM) normalization. Genes were identified as differentially expressed if they had a log_2_ FC greater than or equal to ±1.5 compared to the empty vector and a false-discovery rate (FDR) of <0.05. Additionally, we excluded genes with low expression by requiring at least 3 of the total six individual counts per million (CPM) mapped reads obtained for a given gene to be greater than 2.

### qPCR.

E. coli CFT073 strains harboring pBAD or pBAD-*tosR*-His_6_ were cultured overnight in LB with ampicillin and then diluted 1:100 into fresh LB medium containing ampicillin and cultured to an OD_600_ of 0.15, at which point 10 mM l-arabinose was added to the cultures to induce gene expression. Samples were collected at an OD_600_ of 0.5 to 0.6 and stabilized in phenol-ethanol (95% phenol, 5% ethanol, 4°C). RNA was extracted, and Turbo DNase (Thermo Fisher) was used to eliminate genomic DNA. Removal of genomic DNA was verified by PCR using gapA_f and gapA_r. RNA was converted into cDNA using SuperScript III (Thermo Fisher), and the GenCatch PCR cleanup kit (Epoch Life Sciences) was used to purify cDNA. qPCR was performed using Brilliant III SYBR green master mix (Agilent) with 12 ng of total cDNA. The primers used to detect *papA1*, *papA2*, *aufA*, and *gapA* are listed in Table S4. *gapA* expression was used for normalization of gene expression between samples, and data were analyzed by the threshold cycle (2^−ΔΔ*CT*^) method (99). Data are shown as the log_2_ FC in gene expression compared to CFT073 carrying the empty vector pBAD from three biological replicates.

### Congo red binding assay.

Congo red binding was determined by spotting 5 µl of bacteria cultured overnight in LB medium onto YESCA plates (1 g/liter yeast extract, 10 g/liter Casamino Acids, 20 g/liter agar, 50 µg/ml Congo red [Sigma], 1 µg/ml Coomassie brilliant blue G-250 [Bio-Rad] with 100 µg/ml ampicillin and 10 mM l-arabinose) (100). YESCA plates were incubated at 30°C for 48 h. Congo red binding and RDAR (red, dry, and rough) phenotypes were visually determined using an Olympus SZX16 microscope.

### Calcofluor white binding assay.

To detect cellulose production, 5 µl of overnight culture was spotted onto YESCA plates (1-g/liter yeast extract, 10 g/liter Casamino Acids, 20 g/liter agar, 50 µg/ml fluorescent brightener 28 [calcofluor white, Sigma] with 100 µg/ml ampicillin and 10 mM l-arabinose). Inoculated plates were incubated in the dark at 30°C for 48 h (101). The level of calcofluor white binding to cellulose was visualized using UV light, and images were recorded using a ChemiDoc touch imaging system (Bio-Rad).

### Biofilm formation.

Levels of biofilm formation were quantitatively assessed using crystal violet, modified from reference 102. Briefly, overnight cultures of the CFT073 wild-type or Δ*tosR* and Δ*auf* mutant strains harboring either pBAD, pBAD-*tosR*-His_6_, or pBAD-*aufABCDEFG* were diluted 1:100 into 2 ml salt-free LB medium or human urine with ampicillin and 10 mM l-arabinose in 6-well plates (Cellstar; BioExpress). Plates were incubated statically at 37°C for 24 h. After incubation, unbound cells were removed by washing with water, and the remaining material was stained with 0.1% crystal violet (Fisher) for 15 min. Excess crystal violet was removed by rinsing with phosphate-buffered saline (PBS: 137 mM NaCl, 2.7 mM KCl, 10 mM Na_2_HPO_4_, 1.8 KH_2_PO_4_, pH 7.4) three times followed by resuspension of the retained crystal violet (80:20 ethanol-acetone). OD_590_ was measured with a µQuant plate reader (BioTek).

### Data availability.

The RNA-Seq data discussed in this publication have been deposited in NCBI’s Gene Expression Omnibus repository under accession no. GSE112878.
