# Supplementary figures and images for: TosR-Mediated Regulation of Adhesins and Biofilm Formation in Uropathogenic Escherichia coli
Source: mSphere. 2018 May 16;3(3):e00222-18. doi: 10.1128/mSphere.00222-18 (PMC5956150; doi:10.1128/mSphere.00222-18)

Fig. S1

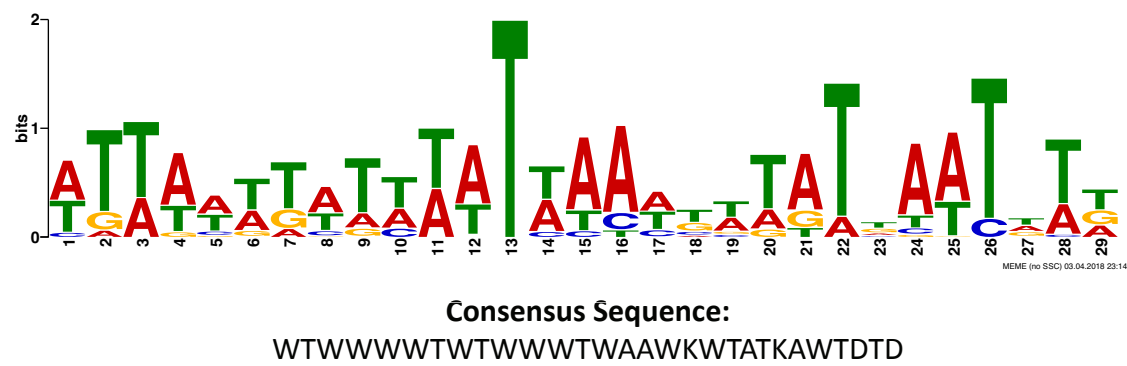

Supplement: FIG S1 [file sph003182550sf1.pdf]

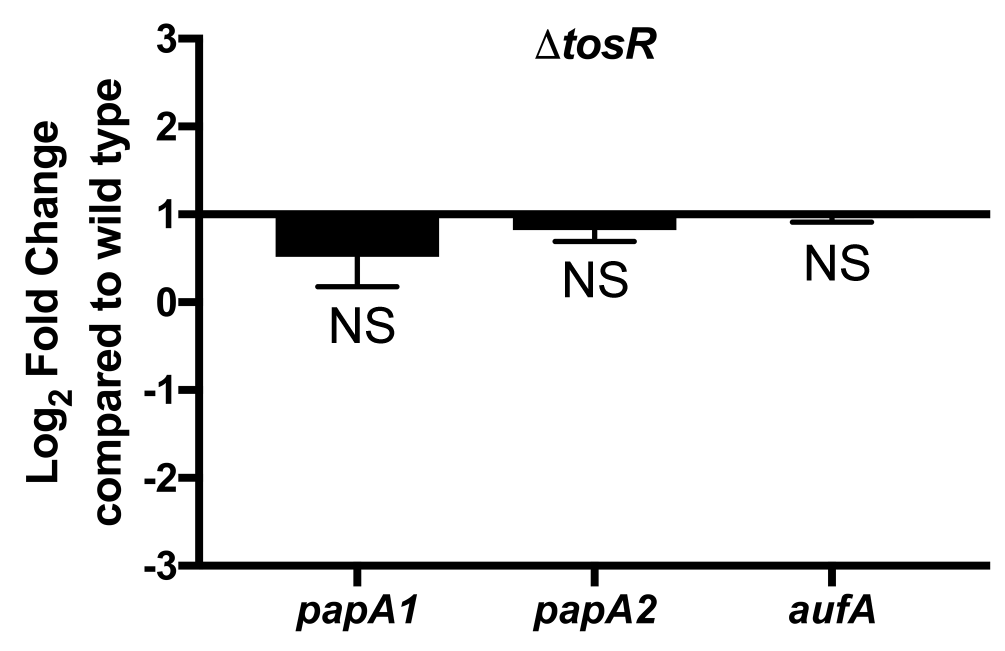

Supplement: FIG S2 [file sph003182550sf2.tif]

Fig. S3

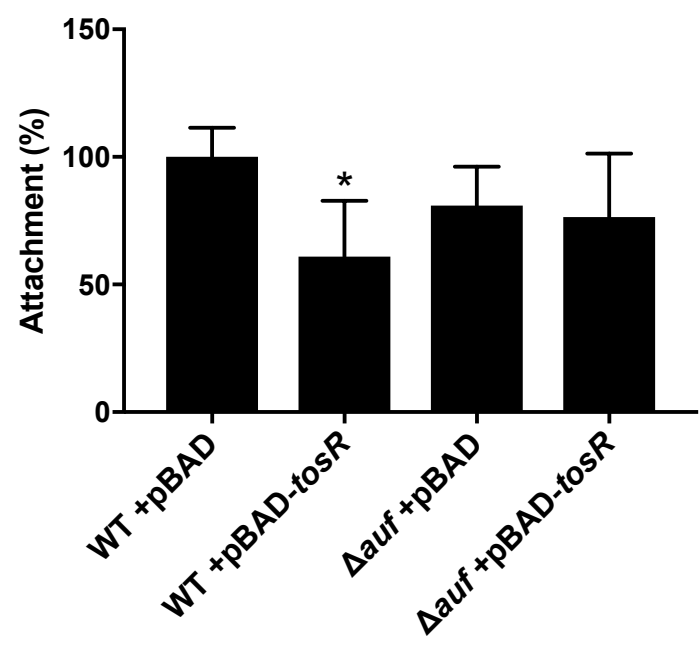

Supplement: FIG S3 [file sph003182550sf3.pdf]

Fig. S4

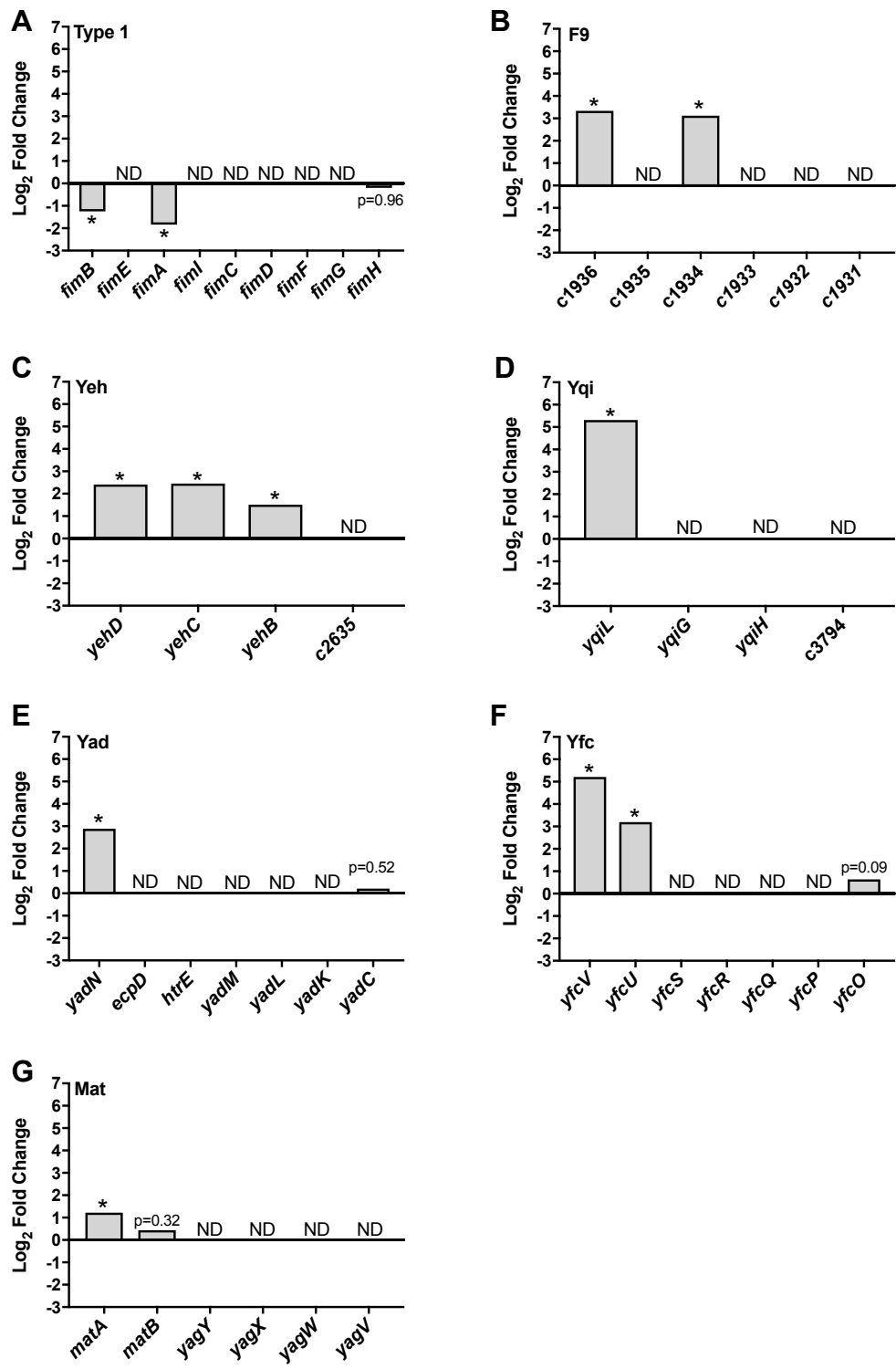

Supplement: FIG S4 [file sph003182550sf4.pdf]

Fig. S5

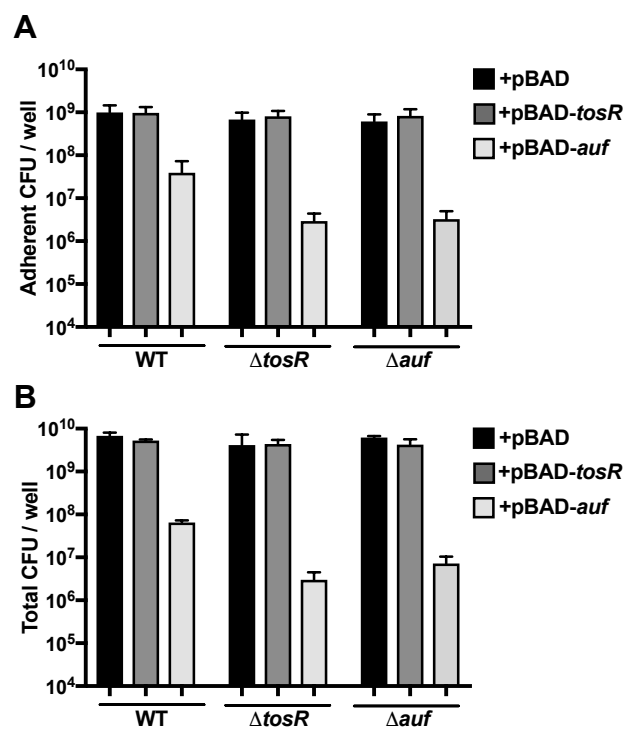

Supplement: FIG S5 [file sph003182550sf5.pdf]
