# Supplementary material for: TosR-Mediated Regulation of Adhesins and Biofilm Formation in Uropathogenic Escherichia coli
Source: mSphere. 2018 May 16;3(3):e00222-18. doi: 10.1128/mSphere.00222-18 (PMC5956150; doi:10.1128/mSphere.00222-18)
Supplement: TABLE S1 [file sph003182550st1.pdf]

Table S1

**Genomic island**[a]	**%G+C**	**Size (kb) of genomic island**	**No. of genes**[b]
PAI-*asnT*	57	32	0
PAI-*metV*	53	32	0
GI-*asnW*	53	54	1
ϕ-*potB*	51	44	1
PAI-*icdA*	50	54	0
GI-*cobU*	50	44	2
ϕ-b0847	50	33	0
PAI-*serX*	49	113	23
ϕ-*smpB*	49	48	4
PAI-*pheU*	48	52	9
PAI-*aspV*	47	100	12
PAI-*pheV*	47	123	16
GI-*selC*	47	68	0

[a]PAI, pathogenicity island - contains known virulence genes; GI, genomic island - contains genes with unconfirmed or no roles in virulence; ϕ, contains phage-rich DNA sequences.
[b]Genes identified by RNA-Seq to be differentially regulated following induction of *tosR.*
