# Supplementary material for: TosR-Mediated Regulation of Adhesins and Biofilm Formation in Uropathogenic Escherichia coli
Source: mSphere. 2018 May 16;3(3):e00222-18. doi: 10.1128/mSphere.00222-18 (PMC5956150; doi:10.1128/mSphere.00222-18)
Supplement: TABLE S2 [file sph003182550st2.pdf]

Table S2

Putative TosR binding motif: WTWWWWTWTWWWTWAAWKWTATKAWTDTD

Top 25 genes upregulated in response to *tosR* overexpression				
**Gene locus**	**Gene name**	**Putative DNA binding sequence (5' → 3')**	**Start[a]**	***P* value[b]**
c0359	*tosR*	AACAATAATATCTATAATATAGATATTAT	254	1.32E-07
c4594	c4594	ATATATTTTATATTTATAGTTTGATTGTT	181	2.40E-06
c0092	c0092	ATTATTGATCAATTAATGTTAAGAATTAA	117	1.42E-08
c4208	*aufF*	TTAATATATACCATTAATATATATTAAAT	3985	2.43E-07
c4924	c4924	CTTATAAAACAAATATATAAATATTTTAT	78	1.23E-06
c4212	*aufC*	TTAATATATACCATTAATATATATTAAAT	1954	2.43E-07
c4210	*aufD*	TTAATATATACCATTAATATATATTAAAT	4659	2.43E-07
c4213	*aufB*	TTAATATATACCATTAATATATATTAAAT	1138	2.43E-07
c4214	*aufA*	TTAATATATACCATTAATATATATTAAAT	360	2.43E-07
c3791	*yqiL*	ATTACTAATTAATAATATAAATTAATAAG	342	4.81E-07
c2884	*yfcV*	TATATTTATTGATTTAATTAATATTAAAT	365	3.46E-08
c4423	c4423	ATAATTAATTATTTAAATTTTTCAATCAG	299	7.40E-07
c3724	*pitB*	-[c]	-	-
c4209	*aufE*	TTAATATATACCATTAATATATATTAAAT	5077	2.43E-07
c4589	*yicP*	-	-	-
c0325	c0325	TTTTTTTCTTAATATCATTAATAAATTTA	97	4.31E-07
c0360	*tosC*	AACAATAATATCTATAATATAGATATTAT	254	1.32E-07
c0322	*efuD*	CATATTGATATAGTTAACTAATTATCTAT	288	3.14E-06
c5444	*yjjQ*	ATTATTTAATTATAAATTAAATGAATGAG	258	1.52E-09
c0323	*efuE*	CATATTGATATAGTTAACTAATTATCTAT	1732	3.14E-06
c2408	c2408	TGTTAGTTATTTTAAAAAATATAAACTTT	71	6.17E-06
c3178	c3178	CAGAATGACACGTTTTATTAATAAATAAA	42	1.17E-07
c1936	c1936	-	-	-
c0435	c0435	-		-
c4894	*tsx*	-	-	-

Top 25 genes downregulated in response to *tosR* overexpression				
**Gene locus**	**Gene name**	**Putative DNA binding sequence (5' → 3')**	**Start**	***P* value**
c1684	*narK*	TTAAATGAGCAATAACCTTAATGAATGTG	282	2.16E-07
c2330	*sdiA*	ATAAATTATATATAAATCTTATTTATGTG	56	2.21E-09
c3655	c3655	-	-	-
c1682	*narX*	CACATTCATTAAGGTTATTGCTCATTTAA	21	2.16E-07
c5187	*papH2*	-	-	-
c3998	*yhcS*	TGTAGATTGATATTTAATATATTAACGTA	58	4.44E-06

c1241	*focC*	CATGTTTACAACATAAAAAACTAAATATA	1971	4.07E-06
c2998	*yffB*	TTTAATGTTATTTAATAGTTGTTAATTTG	100	3.00E-08
c1240	*sfaD*	CATGTTTACAACATAAAAAACTAAATATA	1406	4.07E-06
NA[d]	*papB*	AATATTTACAACATAAAAAACTAAATTTA	67	1.81E-06
NA	*papB2*	-	-	-
c2701	*yeiC*	-	-	-
c5180	*papF2*	-	-	-
c5188	*papA2*	-	-	-
c3733	*hybA*	-	-	-
c3591	*papH*	AATATTTACAACATAAAAAACTAAATTTA	1224	1.81E-06
c1244	*focG*	CATGTTTACAACATAAAAAACTAAATATA	5928	4.07E-06
c5333	*pmbA*	-	-	-
c1245	*focH*	CATGTTTACAACATAAAAAACTAAATATA	6382	4.07E-06
c1246	c1246	CATGTTTACAACATAAAAAACTAAATATA	7696	4.07E-06
c2158	*ynjE*	-	-	-
c1243	*focF*	CATGTTTACAACATAAAAAACTAAATATA	5379	4.07E-06
c1681	*narL*	CACATTCATTAAGGTTATTGCTCATTTAA	1744	2.16E-07
c3584	*papF*	AATATTTACAACATAAAAAACTAAATTTA	6942	1.81E-06
c1239	*focA*	CATGTTTACAACATAAAAAACTAAATATA	778	4.07E-06

[a]No. of bp upstream of ATG of the listed gene.
[b]Site *P* value as determined by Multiple Em for Motif Elicitation (MEME).
[c]–, no consensus sequence identified.
[d]NA, not annotated.
