# Supplementary material for: TosR-Mediated Regulation of Adhesins and Biofilm Formation in Uropathogenic Escherichia coli
Source: mSphere. 2018 May 16;3(3):e00222-18. doi: 10.1128/mSphere.00222-18 (PMC5956150; doi:10.1128/mSphere.00222-18)
Supplement: TABLE S3 [file sph003182550st3.pdf]

Table S3

**Strain**	**Genotype/resistance/use**[a]	**Source**
CFT073	Wild-type pyelonephritis isolate (O6:K2:H1)	1
Δ*tosR*	CFT073 *tosR::kan*	2
Δ*csgD*	CFT073 *csgD::kan*	This study
Δ*aufABCDEFG*	CFT073 *aufABCDEFG::kan*	This study
**Plasmid**	**Relevant characteristic(s)**	**Reference**
pBAD-*myc*-HisA	Vector carrying arabinose inducible *ara*BAD promoter, Amp^r	Invitrogen
pBAD::*aufABCDEFG*	*aufABCDEFG* cloned into the NcoI and HindIII sites of pBAD-*myc*-HisA	This study
pBAD-*tosR*-His_6	*tosR* cloned into NcoI and HindIII sites of pBAD-*myc*-HisA	2
pKD4	Vector carrying a FRT-flanked *kan* gene (Amp^r, Kan^r)	3
pKD46	Vector carrying phage λ Red recombinase, Amp^r	3

[a]kan, kanamycin; amp, ampicillin; r, resistant.

1. Mobley HL, Green DM, Trifillis AL, Johnson DE, Chippendale GR, Lockatell CV, Jones BD, Warren JW. 1990. Pyelonephritogenic *Escherichia coli* and killing of cultured human renal proximal tubular epithelial cells: role of hemolysin in some strains. Infect Immun 58:1281-1289.
2. Engstrom MD, Alteri CJ, Mobley HL. 2014. A conserved PapB family member, TosR, regulates expression of the uropathogenic *Escherichia coli* RTX nonfimbrial adhesin TosA while conserved LuxR family members TosE and TosF suppress motility. Infect Immun 82:3644-3656.
3. Datsenko KA, Wanner BL. 2000. One-step inactivation of chromosomal genes in *Escherichia coli* K-12 using PCR products. Proc Natl Acad Sci U S A 97:6640-6645.
