# Supplementary material for: TosR-Mediated Regulation of Adhesins and Biofilm Formation in Uropathogenic Escherichia coli
Source: mSphere. 2018 May 16;3(3):e00222-18. doi: 10.1128/mSphere.00222-18 (PMC5956150; doi:10.1128/mSphere.00222-18)
Supplement: TABLE S4 [file sph003182550st4.pdf]

**Table S4**

| Primer | Purpose | Sequence (5'→3') |
|---|---|---|
| ΔaufKO_f | Amplification of kanamycin cassette from pKD4 for λ Red recombineering with *aufABCDEFG* | GCTTGGATTTTTTAACAAAAGGAAAGGTATAAATGGTGTAGGCTGGAGCTGCTTC |
| ΔaufKO_r | Amplification of kanamycin cassette from pKD4 for λ Red recombineering with *aufABCDEFG* | TGCGCAGAAGCAGCCTAGTTTTCCACCAATCTGAAATGGGAATTAGCCATGGTCC |
| ΔaufKO_f | Amplification of kanamycin cassette from pKD4 for λ Red recombineering with *csgD* | TGTGCGATCAATAAAAAAAGCGGGGTTTCATCATGGTGTAGGCTGGAGCTGCTTC |
| ΔcsgDKO_r | Amplification of kanamycin cassette from pKD4 for λ Red recombineering with *csgD* | AACGTTTCATGGCTTTATCGCCTGAGGTTATCGTTATGGGAATTAGCCATGGTCC |
| auf_screen_f | Screening for deletion of *auf* operon | AAGAACCTTCTGGAATTAGC |
| auf_screen_r | Screening for deletion of *auf* operon | AGCCAGTGCATTATAACGAC |
| csgD_screen_f | Screening for deletion of *csgD* | GCAACATCTGTCAGTACTTC |
| csgD_screen_r | Screening for deletion of *csgD* | GAAATTCTGCCGCCACAATC |
| pBAD_screen_f | Screening for insertion into pBAD-*myc*-HisA | TGCCATAGCATTTTTATCC |
| pBAD_screen_r | Screening for insertion into pBAD-*myc*-HisA | CTGATTTAATCTGTATCAGG |
| pBAD_auf_f | Amplification of *aufABCDEFG* with 5' NcoI restriction site for cloning into pBAD-*myc*-HisA | NNNNCCATGGCCAAATTCAATTTATCTAATTTATCCGCAG |
| pBAD_auf_r | Amplification of *aufABCDEFG* with 5' KpnI restriction site for cloning into pBAD-*myc*-HisA | NNNNGGTACCCAGGTAAAGTCAGAAAAGTAAC |
| gapA_f | qPCR | CGTTAAAGGCGCTAACTTCG |
| gapA_r | qPCR | ACGGTGGTCATCAGACCTTC |
| papA1_f | qPCR | ATTTGATGGTGCGACAGCAACAGG |
| papA1_r | qPCR | TCTGTTACAGGGTTGCCACTACCA |
| papA2_f | qPCR | CGGGTGAAATTTGATGGAGCCACT |
| papA2_r | qPCR | AGGCACCTTCAGCTACATTCTTGC |
| aufA_f | qPCR | GAATCGGTTGCGACCTTACA |
| aufA_r | qPCR | CAGGCTCACTGATATGGATGAC |
